# Linking Parent–Child and Peer Relationship Quality to Empathy in Adolescence: A Multilevel Meta-Analysis

**DOI:** 10.1007/s10964-019-00993-5

**Published:** 2019-02-27

**Authors:** Savannah Boele, Jolien Van der Graaff, Minet de Wied, Inge E. Van der Valk, Elisabetta Crocetti, Susan Branje

**Affiliations:** 1 Department of Developmental Psychology, Tilburg University, Tilburg, Netherlands; 2 Research Centre Adolescent Development, Utrecht University, Utrecht, Netherlands; 3 Department of Psychology, Alma Mater Studiorum University of Bologna, Bologna, Italy

**Keywords:** Relationship quality, Parent–child relationship, Peer relationship, Empathy, Adolescence, Multilevel meta-analysis

## Abstract

Empathy, which is the ability to feel concern for and to understand others’ feelings, is thought to develop in high quality relationships with parent and peers, but also to facilitate the quality of these relationships. While a wide literature has addressed this aspect, the heterogeneity of primary studies, in which different indicators of relationship quality (e.g., support, conflict) and empathy (i.e., affective and cognitive) have been examined, makes it difficult to draw conclusive answers. Therefore, it remained ambiguous how parent–child and peer relationship quality are associated with adolescents’ empathy. In order to increase the understanding of these associations, a multilevel meta-analysis was performed, which allowed for including multiple effect sizes from each study. By a systematic literate search, 70 eligible studies were found that provided 390 effect sizes from 75 independent samples. The results showed a small positive correlation between parent–child relationship quality and empathy, and a small-to-moderate positive correlation between peer relationship quality and empathy, which was significantly stronger than the correlation with parent–child relationship quality. Hence, the meta-analytic results indicate that adolescents with higher quality relationships, especially with peers, indeed tend to show more concern for and understanding of others’ emotions than adolescents with lower quality relationships. Moreover, the moderation analyses showed stronger correlations for the positive dimension of relationship quality than for the negative dimension, and stronger correlations for composite scores of affective and cognitive empathy than for separate scores of the empathy dimensions. However, no differences in correlations were found between the affective and cognitive empathy dimension, and no moderation effects were found for gender and age. Thus, this meta-analysis demonstrates robust positive associations between parent–child and peer relationship quality and empathy in adolescence, implying that good empathic abilities may be a protective factor for experiencing poor relationships.

## Introduction

Relationship quality and empathy are thought to be closely related in adolescence. High relationship quality is considered essential for the socialization of empathy, which can occur through the modeling of warm and supportive behavior (Barnett 1987; Eisenberg et al. 2003). Reversely, empathy may foster the quality of adolescents’ relationships, as a higher ability to share and understand others’ emotions is related to more prosocial and less aggressive behavior towards others (Eisenberg et al. 2010) and better conflict resolution strategies (e.g., de Wied et al. 2007). Nonetheless, there still exists some uncertainty about the association between relationship quality and empathy in adolescence, because of the variation in research design and inconsistencies in results of empirical studies on this topic thus far. Therefore, this meta-analysis examined concurrent correlations between adolescent relationship quality and empathy in community samples. Relationship quality with parents and peers were separately examined and moderation analyses were conducted to explain differences in strengths of correlations between studies.

### Relationship Quality and Empathy

#### Defining empathy

Empathy is a multidimensional construct that involves other-oriented affective and cognitive responses to another person’s emotions. Affective empathy includes sharing similar emotions (emotional contagion) or feeling sorrow or concern for the observed other (empathic concern). Cognitive empathy refers to the understanding of another person’s feelings, for example through perspective taking or mentalizing processes (Davis 1983). Affective and cognitive empathy are related (e.g., Jolliffe and Farrington 2006; Van der Graaff et al. 2018), indicating that individuals with a better understanding of other’s emotions are likely to also experience more shared feelings or empathic concern. Indeed, it is understood that both responses are needed to enable empathic behavioral responses to others, such as offering comfort (Davis 1983). Although they are connected, affective and cognitive empathy are also distinct processes (Decety and Jackson 2004), and previous research revealed different associations with social functioning (e.g., Batanova and Loukas 2011; Van der Graaff et al. 2018). Thus, although affective and cognitive empathy are conceptually and empirically interrelated, they also tap into different aspects of empathy, and it is important to examine their effects separately, as associations with relationship quality may be different. It was therefore assessed how parent–child and peer relationship quality were uniquely associated with affective and cognitive empathy, as well as how parent–child and peer relationship quality were associated with a combination of both.

#### Socialization of empathy

Parents are considered to have an important role in the socialization of empathy (Hoffman 2000). According to the social learning perspective, warm and supportive parents model empathy because they consider their child’s perspective and show concern for their emotions (Barnett 1987). The attachment theory posits that warm and supportive parenting satisfies children’s emotional needs, which can lead to less preoccupation with one’s own emotions and therefore a better ability to attend to the other person’s emotions (Bowlby 1982). Moreover, negative parent–child interaction, such as parental hostility and parent–child conflicts, might impede children’s emotional regulation skills and hence may hinder perspective taking and showing concern for others (Morris et al. 2007). Although evidence for the role of parents in empathy development comes mainly from studies in childhood, these parental socialization processes are thought to still take place in adolescence.

Importantly, socialization through peer relationships should also be considered, as adolescents become more independent from their parents and spend increasingly more time with peers (Smetana et al. 2006). As peer relationships are particularly characterized by high levels of equality, intimacy, and trust (Youniss and Smollar 1985), the high frequency of intimate interactions provide ample opportunities to observe and model warm and supportive behavior, which may facilitate the development of affective empathy. However, interactions with peers are thought to be essential for specifically the development of cognitive empathy. That is, through reciprocal sharing of thoughts and feelings, disagreements between peers are assumed to facilitate the ability to take others’ point of view (Selman 1980), a capability that is still developing in adolescence (Van der Graaff et al. 2014).

Although parental socialization of empathy is expected to continue in adolescence, the contribution of peer socialization on adolescents’ empathy development empathy might be larger. An important difference between the relationships is that adolescents form new, voluntary peer relationships, which is in contrast to their permanent, involuntary relationship with their parents (Laursen and Bukowski 1997), and which makes it more necessary for adolescents to understand their peer’s perspective. Peer relationships are also more horizontal than parent-adolescent relationships, with more egalitarian interactions that are thought to facilitate perspective taking (Selman 1980). Relatedly, during adolescence, parent–child relationships temporarily become less supportive (De Goede et al. 2009a), whereas peer relationships become more supportive (De Goede et al. 2009b). Additionally, in general, peer relationships are more intimate and reciprocal than parent–child relationships (Laursen and Bukowski 1997), and hence co-rumination about each other’s problems is a typical aspect of close peer relationships (Rose 2002). Thus, having frequent reciprocal, intimate, and supportive interactions with like-minded persons is considered to be an important ingredient in the development of empathy (Selman 1980; Youniss 1980).

#### Role of empathy in relationships

While socialization theories provide the most common background for the association between relationship quality and empathy, it is likely that one’s ability for empathy also affects the quality of relationships with others. When persons can share and understand feelings of others, it allows them to meet others’ needs – thus, permitting them to be supportive and to solve conflicts with compromises. Indeed, the results of prior research suggest that higher levels of empathy predicted more supportive relationships with parents (Miklikowska et al. 2011) and peers (Smith and Rose 2011), and better problem solving capacities in conflicts with parents (Van Lissa et al. 2016). When comparing parent–child and peer relationships, empathy may be particularly important to gain and maintain high quality relationships with peers. Despite average changes in interaction patterns between children and parents during adolescence (Furman and Buhrmester 1992; Larson and Richards 1991), the quality of the parent–child relationship remains relatively continuous, as it is an accumulation of previous experiences in childhood (Laursen et al. 2010). Relationships with peers, however, are being formed and increasingly fulfill functions of closeness, supportiveness, and intimacy during adolescence (Furman and Buhrmester 1992; Larson and Richards 1991). Thus, it was expected that adolescents with high levels of empathy have high quality relationships, particularly with peers.

### The Need for a Meta-Analysis

Even though the theoretical background implies a positive association between adolescent relationship quality and empathy, a meta-analysis is needed for several reasons. First, empirical results show inconsistencies. For example, different correlations have been reported for boys and girls (e.g., Adams et al. 1982; Heller et al. 2007), for affective and cognitive empathy (Li et al. 2015; Meuwese et al. 2017), and between different informants for relationship quality (Soenens et al. 2007). Second, researchers have examined relationship quality with a divers set of indicators, ranging from overall quality (Smith and Rose 2011) and satisfaction (Haugen et al. 2008; Sillars et al. 2005), to specific indicators, such as support (de Kemp et al. 2007), open communication (Heller et al. 2007), and conflict frequency (Van Lissa et al. 2015). Given the empirical inconsistencies and variation in research designs, a meta-analysis can present a better overview by providing an overall correlation and by considering potential moderators to increase the understanding of the heterogeneity among results of previous studies.

#### Dimension of relationship quality

A distinction was made between the positive (e.g., support) and negative dimension (e.g., conflict) of relationship quality to test whether they were differently related to empathy in adolescence. With respect to parent–child relationship quality, a positive association between the positive dimension of relationship quality and empathy was expected. However, concerning the negative dimension of parent–child relationship quality, it was expected that the association with empathy could go in both directions. On the one hand, more frequent conflicts with parents in which parents explain their point of view might enhance perspective-taking abilities, and therefore adolescents with more parent–child conflicts may score higher on empathy, resulting in a positive association. On the other hand, adolescents with better perspective taking abilities might have less conflicts with their parents or might be less likely to show maladaptive conflicts resolution styles, because they have a better understanding of the emotions of their parents and can respond more empathically (Eisenberg et al. 2010), which would result in a negative association.

Concerning peer relationship quality, a positive association was expected between the positive dimension of relationship quality and empathy, and the direction of the association with the negative dimension of peer relationship quality can also be theorized in both directions. First, (higher frequency of) peer conflicts can be positively associated with empathy, as disagreements between peers are thought to actually facilitate perspective taking because of reciprocal sharing of perspectives (Selman 1980; Youniss 1980). Second, peer conflicts and in particular poor conflict resolution strategies can be negatively associated with empathy, because higher perspective taking may facilitate constructive conflict solving (De Wied et al. 2007), and because strong negative emotions might be so overwhelming that they prevent the adolescent to attend to the perspective of the other.

#### Dimension of empathy

Even though affective and cognitive empathy are seen as conceptually and empirically related (Decety and Jackson 2004; Van der Graaff et al. 2018), different associations with parent–child and peer relationship quality can be expected based on socialization theories. That is, parental socialization theories specifically highlight the important role of parental warmth and support in the development of affective empathy (Hoffman 2000), whereas theories about peer socialization place more emphasis on the role of reciprocity and mutual understanding between peers in the development of perspective taking (Selman 1980). Therefore, it was expected that parent–child relationship quality is more strongly associated with affective empathy than to cognitive empathy, and peer relationship quality is more strongly associated with cognitive empathy than to affective empathy.

#### Type of relationship

With respect to the parent–child relationship, it has been suggested that the role of fathers in children’s prosocial development is less substantial than the role of mothers, as fathers have less opportunities to reinforce and support children’s prosocial development, because they spend less time with their children (Yeung et al. 2001), and are less aware of their children’s prosocial behavior (Hastings et al. 2007). However, there is also evidence indicating that fathers are more involved in the socialization of cognitive empathy and mothers in affective empathy (Miklikowska et al. 2011). To clarify this, it was examined whether mother- and father-relationship quality are differently related to adolescent cognitive and affective empathy.

Regarding peer relationships, a distinction was made between quality of relationships between friends, romantic partners, and siblings. Socialization influences in these relationships can vary in strength, because friendships and romantic relationships are voluntary and mainly based on equality and reciprocity, whereas sibling relationships are involuntary and may be less equal and reciprocal due to age differences (Laursen and Bukowski 1997). Hence, it was expected that the association of empathy with friendship and romantic relationship quality is stronger than the association of empathy with sibling relationship quality.

#### Age

It was examined whether the correlation between relationship quality and empathy varied as a function of age. As adolescents gain more independence from their parents and spend less time with them (Smetana et al. 2006), it was expected that the association between parent–child relationship quality and adolescents’ empathy is stronger for younger than older adolescents. In contrast, because time spent with peers and intimacy between peers increases during adolescence (Buhrmester and Furman 1987; Larson and Richards 1991), it was expected that the association between peer relationship quality and empathy is stronger for older than younger adolescents.

#### Gender

Gender differences were expected in the correlations, as parents might socialize girls and boys differently according to gender role expectations (e.g., girls are expected to be more affectionate and caring than boys) (Bem 1981; Kennedy Root and Denham 2010). Especially in adolescence, when puberty sets in and physical gender differences become apparent, gender stereotypical behavior might be more reinforced within the parent–child relationship. Hence, parents might act as better empathic models for and encourage more empathic behavior in adolescent girls than boys. Furthermore, within peer relationships, girls tend to focus more on cooperation, whereas boys focus more on status and competition (Rose and Rudolph 2006). Therefore, the positive loop between peer relationship quality and empathy might be stronger for girls than boys, because higher levels of empathy in girls are likely to promote more cooperation and mutual support, and vice versa. Thus, it was expected that the correlation of both parent–child and peer relationship quality with empathy is stronger for girls than for boys, particularly regarding affective empathy.

#### Additional sample, study, and measurement characteristics

In addition to the above-mentioned moderators, the following moderators without specific hypotheses were explored: ethnic composition of the sample, publication year, reliability of measures, informants, and assessment method.

## The Present Study

The aim of the present study is to increase the understanding of how parent–child and peer relationship quality are related to empathy in adolescence, because empirical studies vary in their research designs (e.g., cognitive versus affective empathy, support versus conflict), and because to date, no synthetization was available. To this aim, concurrent correlations of adolescent parent–child and peer relationship quality with empathy in a community population were assessed with meta-analytic models. Based on socialization theories (Hoffman 2000) and the implication that empathy fosters high quality interaction (e.g., Van Lissa et al. 2016), positive associations were expected between parent–child and peer relationship quality and empathy in adolescence. Additionally, as peer relationships are considered to play a key role in social development (Youniss 1980), the correlation between peer relationship quality and empathy was expected to be stronger than the correlation between adolescent parent–child relationship quality and empathy. To examine these hypotheses, a multilevel approach was adopted, allowing to include multiple effect sizes from one study or sample (Van den Noortgate et al. 2015). In addition, moderator analyses were conducted to explain inconsistent results available in the literature by showing which factors might amplify or attenuate the concurrent correlations.

## Method

### Study Retrieval

#### Search strategy

The initial search was performed in November 2016 and updated in October 2018 and January 2019. Eligible studies were searched for in electronic databases: PsycINFO, PsycARTICLES, Psychology and Behavioral Sciences Collection, ERIC, and Web of Science Social Sciences Citation Index (SSCI). The search was constrained to peer-reviewed articles written in English. Studies had to use at least one key word in the title, keywords, and/or abstract for each of the following aspects: (I) relationship quality, (II) type of relationship, (III) empathy, and (IV) adolescents.

I. Key words for overall relationship quality (i.e., “relation* quality” or satisfact*) and parent–child or peer relationship (i.e., “parent-adolescent relation*” or “parent–childrelation*” or “*friendship*” or “peer relation*” or “sibling relation*”). Separate key words were used for the positive dimension (i.e., attach* or bond* or close* or *proximity* or cohesi* or intima* or secur* or harmon* or companion* or *alliance* or cooperat* or affect* or support* or nurtur* or *warmth* or *admiration* or trust* or loyal* or communicat* or “*positive affect”* or “*positive interaction*” or equal* or *egalitarian* or *symmetric*) and negative dimension (i.e., conflict* or *disagreement* or argue* or bicker* or fight* or agressi* or hostil* or *alienation* or “*negative affect”* or “*negative interaction”* or inequal* or *asymetric* or *power* or dominan* or authorit*).
II. Key words for type of relationship: mother* or *maternal* or father* or *paternal* or parent* or *family* or peer* or friend* or partner* or sibling* or *brother* or *sister* or *twin* or *relative*.
III. Key words for empathy: empath* or sympath* or “vicarious emotional respond*” or “emotion* *contagion*” or “*theory of mind*” or (“*compassion*” not “*self-compassion*”) or “*perspective taking*” or “*role taking*” or mentaliz* or “emotion* *recognition*”).
IV. Key words for adolescence: adolescen* or *youth* or teen* or “*middle school*” or “*high school*” or “*secondary school*” or “*young people*”.

To exclude studies based on clinical samples a subsequent search string was used: NOT autis* or disorder* or diagnos* or disabilit* or *syndrome* or *disease* or *illness*.

In addition to the database search, references of eligible studies were manually checked in the reference lists of selected studies (i.e., backward citation search) in order to identify articles that were missed in the database search.

#### Inclusion criteria

Studies were included when they met the following four criteria. First, the study had to assess overall relationship quality, or indicators of the positive (i.e., warmth and support) or negative dimension (i.e. conflict). Although key words were used for the dimension of power, correlations between indicators of power and empathy were excluded, because indicators of power imbalance within the parent–child relationship overlap with parenting practices (e.g., parental control and monitoring) and are therefore less applicable to peer relationships (Furman 1998). When a composite score of relationship quality also comprised power imbalance, the study was not excluded, but it was coded that the measurement also included power imbalance. Consequently, the effect size with power imbalance was included when calculating the overall correlation but was excluded when correlations were calculated for a specific relationship quality indicator (e.g., warmth, conflict). Moreover, prosocial behavior within relationships was not included as an indicator of the positive dimension, as it is conceptually too closely related to empathy (Eisenberg and Fabes 1990). Furthermore, the aim was to examine the stable quality of the relationship, and thus studies that manipulated relationship quality (e.g., induced conflict situations) were excluded.

Second, the study had to assess relationship quality with parents or peers. Peers could include peers in general (when authors did not further specify what sort of peer it involved, such as friends or classmates), friends, romantic partners, and siblings. Siblings were considered as peers if both siblings were adolescents (age between 10–20 years). Furthermore, studies that examined relationship quality with family members in general were excluded, because this might also include siblings and/or grandparents.

Third, empathy had to be an affective or cognitive response to another person’s emotions (or a combination). Affective responses included emotional contagion and empathic concern. Cognitive responses included empathic accuracy, perspective taking, and mentalizing. Hence, personal distress and prosocial behavior were excluded, because personal distress is a *self-oriented* affective response and prosocial behavior is a *behavioral* response (Davis 1983).

Fourth, studies had to assess a community sample with mean age between 10 and 20 years old. Clinical samples were excluded, because levels of relationship quality and empathy may be non-normative in such samples.

### Study Selection

**Fig. 1:**
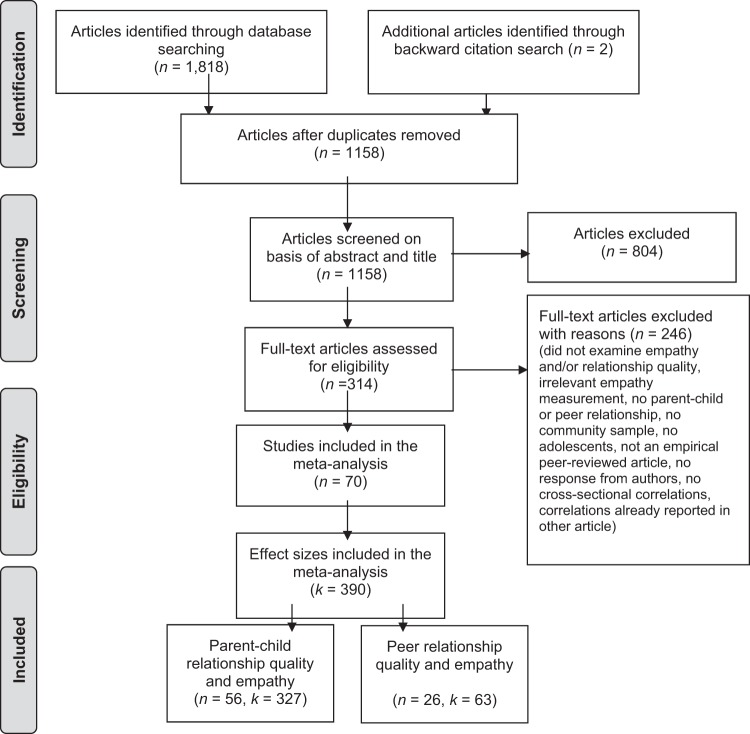
PRISMA flow diagram of the systematic search. Note. *n* = number of studies. *k* = number of correlations

The database search resulted in 1158 unique hits. The second author screened 10% of the hits and showed an acceptable agreement of *κ* = 0.70 with the first author. Disagreement was solved through discussion and led to improved inclusion and exclusion criteria. Based upon screening of titles and abstracts, the first author retrieved the full text of 314 articles, of which 68 articles were included (see Fig. 1 for exclusion reasons). Two additional studies were found by backward citation search. In sum, 70 eligible studies were found. After clustering studies that analyzed the same sample or separating subsamples within studies, 75 independent samples remained, which had combined 390 correlations. Included studies and their characteristics are reported in Table 1.

**Table 1:** Overview of Included Studies

Study	Snr	*k*	*N*	*M*_age_	% girls	Relationship person(s)	Dimension RQ	Indicator RQ	Informant RQ	Dimension empathy	Indicator empathy	Informant empathy
Adams et al. (1982)	1 2	23	57/58	13 to 18 years^a^	100/0%	Mother, father	Positive	Support, physical affect, companionship	A	Affective, cognitive	Composite affective, empathic accuracy^d^	A, O
Antonopoulou et al. (2012)	3	2	190	11.3	45%	Father	Positive, negative	Warmth and support, verbal hostility	A	Affective	Composite affective^b^	A
Batanova and Loukas (2012)	4 5	4	263/224	11.7	100/0%	Parents	Negative	Conflict frequency	A	Affective, cognitive	Empathic concern, perspective taking	A
Batanova and Loukas (2014)	4 5	4	260/221	11.7	100/0%	Parents	Positive	Composite positive	A	Affective, cognitive	Empathic concern, perspective taking	A
Black and Pedro-Carrol (1993)	6	2	286	18.8	59%	Mother, father	Positive	Emotional security	A	Affective	Composite affective	A
Carlo et al. (2007)	7	1	233	16.7	69%	Parents	Positive	Responsiveness	A	Affective	Composite EC/PT	A
Carlo et al. (2011)	8	6	730	10.8	49%	Mother, father	Positive	Warmth	A	Affective	Composite affective^b^	A
Carlo et al. (1998)	9	2	80	14.2	51%	Mother, father	Positive	Support	P	Affective	Composite EC/PT	A
Carlo et al. (1999)	10	3	89	16.0	52%	Parents	Positive	Attachment	A	Affective, cognitive	Composite EC/PT, empathic concern, perspective taking	A
Carlo et al. (2018)	11	4	306	15.5	46%	Parents, friend	Positive	Support	A	Affective, cognitive	Empathic concern, perspective taking	A
Chow et al. (2013)	12	16	151	15.9	49%	Friend	Positive, negative	Closeness, discord^c^	A, F	Affective, cognitive	Empathic concern, perspective taking	A, F
Christensen et al. (2011)	13	4	334	13.2	49%	Mother, father	Positive	Forgiveness	A, P	Affective	Empathic concern	A
Courtain and Glowacz (2018)	1415	4	583/230	18.9	100/0%	Partner	Negative	Positive conflict resolution style	A	Affective, cognitive	Empathic concern, perspective taking	A
Curcio et al. (2017)	16 17 18	3	208/126/228	(13.5/16/19)	—	Parents	Positive	Attachment^c^	A	Affective	Empathic concern	A
Daniel et al. (2016)	19 20	4	683/575	15.3	100/0%	Friend	Positive	Composite positive	A	Affective	Empathic concern	A, P
De Kemp et al. (2007)	21	3	823	12.8	51%	Parents	Positive	Support	A	Affective	Composite aff	A
De Los Reyes et al. (2013)	22	1	50	15.4	60%	Parents	Negative	Conflict^f^	A	Affective	Mentalizing^e^	A
Estevez Lopez et al. (2018)	23	6	1510	13.4	48%	Mother, father	Positive, negative	Communication styles	A	Affective	Composite affective responses^b^	A
Furlong et al. (2017)	24	1	1413	20.0	64%	Friend	Positive	Support	A	Composite	Composite affective and cognitive empathy	A
Green et al. (2018)	25	2	819	11 to 16 years^a^	—	Mother	Positive	Trust	A	Affective, cognitive	Emotional matching, emotional understanding	A
Haugen et al. (2008)	26 27	4	408	17.0	50%	Partner	Positive	Relationship satisfaction	A, RP	Cognitive	Empathic accuracy^e^	A
Heller et al. (2007)	28	4	236	14.8	—	Mother, father	Positive	Open communication	A	Affective, cognitive	Empathic concern, perspective taking	A
Henry et al. (1996)	29	4	149	14.8	51%	Parents	Positive	Warmth and support, communication effectiveness	A	Affective, cognitive	Empathic concern, perspective taking	A
Henry et al. (2005)	30	6	72	15.0	63%	(Step)parents	Positive	Support	A	Affective	Empathic concern	A
Humfress et al. (2002)	31	2	70	12.7	49%	Mother	Positive, negative	Warmth, conflict	A	cognitive	Mentalizing^d^	O
Hünefeldt et al. (2013)	32	8	402	16.7	42%	Mother, father, friend, partner	Negative	Attachment anxiety, attachment avoidance	A	Cognitive	Mentalizing^e^	A
Ingoglia et al. (2011)	33	14	331	17.4	54%	Parents	Positive, negative	Emotional closeness, open communication, mutuality, rejection, open confrontation, distrust, alienation	A	Affective, cognitive	Empathic concern, perspective taking	A
Johnson et al. (2013)	34	1	161	14.7	41%	Friend	Positive	Commitment	A	Affective	Composite affective ^b^	A
Kenny and Gallagher (2002)	35 36	4	97/75	10^th^ and 12^th^ grade (15.5 y)	100/0%	Mother, father	Positive	Attachment	A	Affective	Composite affective	A
Laghi et al. (2009)	37	2	2665	17.3	50%	Parents, peer	Positive	Attachment	A	Composite	Composite EC/PT	A
Laible (2007)	38	2	117	19.6	56%	Parents, peer	Positive	Attachment	A	Composite	Composite EC/PT	A
Laible and Carlo (2004)	39	2	109	16.1	56%	Mother, father	Positive	Support	A	Composite	Composite EC/PT	A
Laible et al. (2000)	40	2	89	16.1	48%	Parents, peer	Positive	Attachment	A	Composite	Composite EC/PT	A
Laible et al. (2004)	41	2	246	18.6	70%	Parents, peer	Positive	Attachment	A	Composite	Composite EC/PT	A
Lam et al. (2012)	42	4	201	10.3/12.9		Mother, father	Positive	Warmth, parental responsiveness, conflict	A	Affective	Affective empathy	A
Lereya et al. (2016)	43	1	7052	12.8	58%	Peer	Positive	Support	A	Composite	Composite affective and cognitive responses	A
Li et al. (2015)	44 45	8	3199/3102	13.1	100/0%	Mother, father	Positive	Attachment	A	Affective, cognitive	Empathic concern, perspective taking	A
Llorca-Mestre et al. (2017)	46	2	610	9 to 128 years (10.5)	52%	Friend	Positive	Attachment	A	Affective, cognitive	Empathic concern, perspective taking	A
Marshall et al. (2001)	47	2	45	15 to 16 years (15.5 y)	56%	Mother, father	Positive	Open communication	A	Affective	Empathic concern	A
McGinley (2018)	48	4	187	18.8	49%	Mother, father	Positive	Attachment	A	Affective, cognitive	Empathic concern, perspective taking	A
McLaren and Pederson (2014)	49	1	190	14.8	65%	Parents	Positive	Closeness	A	Cognitive	Mentalizing^d^	O
Meuwese et al. (2017)	50	4	205	14.4	54%	Friend	Positive, negative	Positive friendship quality, negative friendship quality^c^	A	Affective, cognitive	Emotional contagion, emotional understanding	A
Miklikowska et al. (2011)	51	12	678	15.6	50%	Mother, father	Positive	Responsiveness	A	Affective, cognitive	Empathic concern, perspective taking	A
Miklikowska and Hurme (2011)	52	2	1341	16.9	56%	Parents	Positive	Warmth, open communication	A	Composite	Composite EC/PT	A
Murphy et al. (2015)	53	1	148	15.7	67%	Parents	Positive	Attachment	A	Affective	Empathic concern	A
Nickerson et al. (2008)	54	2	105	12.2	64%	Mother, father	Positive	Attachment	A	Affective	Empathic concern	A
O’Connor and Hirsch (1999)	55	1	39	13.0	44%	Friend	Positive	Friendship quality	A	Cognitive	Mentalizing^f^	A
Oliva et al. (2014)	56	2	2400	14.7	56%	Parents, peer	Positive	Composite positive indicators, peer attachment	A	Composite	Composite affective and cognitive responses	A
Overgaauw et al. (2017)	57	2	862	—	50%	Friend	Positive	Composite positive indicators	A	Affective, cognitive	Emotional matching, emotional understanding	A
Padilla-Walker et al. (2011)	13	6	500	11.5	49%	Mother	Positive	Attachment, connectedness, involvement	A, P	Affective	Empathic concern	P
Padilla-Walker et al. (2015a)	13	4	491	11.5	50%	Mother	Positive	Warmth	A, M	Affective	Empathic concern	A, P
Padilla-Walker et al. (2015b)	13	3	467	13.3	49%	Mother, friend	Positive	Friend connection, friend companionship, maternal warmth and support	A	Affective	Empathic concern	A
Sillars et al. (2005)	58	6	50	11 to 14 years (12.5 y)	44%	Parents	Positive	Relationship satisfaction	A	Cognitive	Empathic accuracy^e^	O
Siu and Shek (2005)	59	2	1426	13.8	60%	Mother, father	Negative	Conflict	A	Composite	Composite EC/PT	A
Smith and Rose (2011)	60	1	208	12.9	58%	Friend	Positive	Friendship quality	A	Cognitive	Perspective taking	A
Soenens et al. (2007)	61	6	162	16.9	51%	Mother, friend	Positive	Maternal support, friendship quality	A, M	Affective, cognitive	Empathic concern, perspective taking	A
Stevens and Hardy (2011)	62	2	310	16.0	60%	Mother, father	Negative	Disrespect	A	Affective	Empathic concern	A
Telef and Furlong (2017)	63	1	2242	16.2	54%	Friend	Positive	Support	A	Composite	Composite EC/PT	A
Thompson and Gullone (2008)	64	1	281	14.8	60%	Parents	Positive	Attachment	A	Affective	Composite affective^b^	A
Van der Graaff et al. (2016)	65	4	379	17.0	44%	Parents	Positive, negative	Support, negative interaction	A	Affective	Empathic concern	A
Van der Graaff et al. (2012)	66	1	323	14.3	51%	Parents	Positive	Support	A	Affective	Affective empathy	A
van Lissa et al. (2015)	65	48	444	13.3	43%	Mother, father	Negative	Conflict	P	Affective, cognitive	Empathic concern, perspective taking	A
van Lissa et al. (2016)	65	88	448	13.0	43%	Mother, father	Positive, negative	Conflict resolution styles	A	Affective, cognitive	Empathic concern, perspective taking	A
Vieira (2015)	67	2	253	11.3	100%	Parents	Positive	Open communication	A	Affective, cognitive	Empathic concern, perspective taking	A
Winstanley et al. (2002)	68	1	59	18.2	0%	Partner	Positive	Intimacy achievement^f^	A	Composite	Composite EC/PT^b^	A
Wu et al. (2015)	69	1	1029	14.5	51%	Parents	Positive	Warmth	A	Composite	Composite EC/PT	A
You and Kim (2016)	70 71	4	282/448	15.2	100/0%	Parents, peer	Positive	Attachment	A	Composite	Composite EC/PT	A
You et al. (2015)	72 73	4	335/401	7^th^ to 9^th^ grade (12.4 y)	100/0%	Mother, friend	Positive	Attachment	A	Composite	Composite affective and cognitive responses	A
Zhang et al. (2013)	74	1	1297	8^th^ and 9^th^ grade (14.4 y)	47%	Peer	Positive	Support	A	Cognitive	Empathic accuracy	A
Zhou et al. (2002)	75	4	169	11.4	50%	Parents	Positive	Warmth and support^d^	O	Affective	Emotional contagion^e^	A, O

Imputed mean age based on reported information is between brackets indicate. Mean age of studies with multiple waves is mean age of the first wave

*Snr* sample (different numbers indicate different samples), *k* number of effect sizes, *N* sample size, *M*_*age*_ mean age, *RQ* relationship quality, *Composite EC/PT* combination of empathic concern and perspective taking, *A* adolescent, *P* parent, *M* mother, *F* friend, *O* observant, *RP* romantic partner

^a^mean age not imputed because of the wide range

^b^included personal distress

^c^included power imbalance

^d^observation

^e^task

^f^interview

### Coding the Studies

The coding scheme documented information of the relationship quality indicator, empathy indicator, study and sample characteristics, and correlation(s). The first author coded all studies and the second author coded a subset of 48 correlations (12%) of 10 studies (14%). For study characteristics, inter-coder reliability was calculated for sample size (ICC = 0.995), mean age (ICC = 1.0), percentage girls (ICC = 0.99), and ethnicity (*κ* = 1.0). For effect size characteristics, inter-coder reliability was calculated for dimensions of relationship quality and empathy (*κ*’s = 1.0), type of parent–child or peer relationship (*κ* = 0.97), and correlation coefficient (ICC = 0.99).

#### Relationship quality

First, it was coded whether the study involved parent–child or peer relationship quality, gender of parent (i.e., mother or father), and type of peer relationship (i.e., peers in general, friend, romantic partner, or siblings). Next, indicators were categorized into the positive and negative dimensions of relationship quality. Higher scores on the positive dimension represented higher relationship quality, such as warmth, support, and constructive conflict resolution style. Indicators of the negative dimension included scales where a higher score reflected lower relationship quality, such as hostility, conflict frequency and poor conflict resolution style. In order to conduct moderator analyses on the complete data, the correlations of the negative dimension were reverse coded, such that all correlations indicated that higher relationship quality was related to higher empathy. Last of all, positive relationship indicators were categorized into three (or four) categories: warmth and support, attachment, (friendship quality,) and communication quality. Not enough variation was present to distinguish between categories of negative relationship indicators. Moreover, assessment method, informant(s), and reliability of the relationship quality measurement were coded. When authors did not report the exact reliability for separate subscales, for example it was not reported for the subscales, the average reliability was coded.

#### Empathy

It was coded whether empathy comprised affective empathy, cognitive empathy, or a composite score that included both affective and cognitive empathy (e.g., sympathy). Affective empathy measures were categorized as empathic concern, emotional contagion, or a combination of affective empathy-related responses. Cognitive empathy measures were categorized as perspective taking, empathic accuracy, or mentalizing. Similar to the approach for relationship quality, assessment method, informant(s), and reliability were coded.

#### Study and sample characteristics

Regarding study characteristics, publication year, country of leading author, and continent of data collection were coded. Regarding sample characteristics^1^, it was coded whether the sample belonged to a specific research project (to detect double samples), sample size, mean age^2^, gender composition (i.e., percentage of girls), and ethnic composition. Ethnic composition was coded as majority ( > 75% of participants’ ethnicity can be considered to be in the ethnic majority in the country of data collection), minority (i.e., when > 75% of participants’ ethnicity can be regarded as the ethnic minority in the country of data collection), or mixed (i.e., when less than 75% of the sample were the ethnic majority or minority).

#### Effect size

Pearson’s product-moment correlation coefficient *r* was chosen as effect size. A small, moderate, and large effect is indicated by *r* = 0.10, 0.30, and 0.50, respectively (Cohen 1992). When authors reported that the correlation was not significant without reporting the exact coefficient, the correlation was coded as zero.

#### Missing information

Of three publications, authors did not respond to the e-mail request to send the full text. Therefore, these potential eligible studies could not be included. If essential information (e.g., mean age, or the target correlation) was not reported in a potential eligible study, authors were contacted through email (with two reminders in absence of a response) with a request to send additional information. In total, thirty requests were sent, of which fifteen requests were met. Studies could not be included when authors did not respond on a request for correlational data (*n* = 13).

### Statistical Analysis

#### Individual effect sizes

Correlation coefficients were transformed to the normally distributed Fisher’s *z*. The Fisher’s *z* score and its variance were used in the meta-analyses, and afterwards back transformed to Pearson’s correlation coefficients. Fisher’s *z* scores were transformed to Pearson’s correlations with the R package “compute.es” (AC Del Re 2013).

#### Overall effect sizes and moderators

A multilevel approach was adopted, which takes into account the hierarchical structure of the dataset, such that effect sizes are nested within studies (Assink and Wibbelink 2016). Hence, a multilevel approach allowed for extracting multiple effect sizes from the same study (e.g., when a study provided separate effect sizes for affective and cognitive empathy) while taking into consideration the dependency between them. Therefore, the multilevel approach ensures that studies with multiple effect sizes do not have a larger influence on the meta-analytic estimates than studies that provided a unique effect size. To control for this dependency, a three-level structure was assigned to the meta-analytic models, which takes into account three different variance components: (1) sampling variance (i.e., variance at the level of the individual effect size), (2) within-study variance (i.e., variance of effect sizes within the same study), and (3) between-study variance (i.e., variance of effect sizes between different studies) (Van den Noortgate et al. 2015).

The statistical analyses were conducted by using the package “metafor” (Viechtbauer 2010) in the statistical software environment R 3.5.0 (R Core Team 2016). Restricted Maximum Likelihood Estimation (REML) was used to reduce bias of variance estimates (Van den Noortgate et al. 2015). First, overall correlations for the associations of parent–child and peer relationship quality with empathy were estimated. Second, heterogeneity in correlations within and between studies was tested. To examine heterogeneity within studies (level 2), it was tested whether the three-level model fitted better than a two-level model (i.e., only sampling and between-study variance) as indicated by a significant one-sided log-likelihood-ratio test, showing that variance is present at the second level. Similarly, heterogeneity between studies was tested by examining the fit of the three-level model against a model that only had variance at the first (sampling variance) and second level (within-study variance). By using the procedure formulated by Cheung (2014), the distribution of variance at each level of the model was calculated.

If the results indicated heterogeneity, moderator analyses were conducted to explain it. Variables were tested as moderators when data were available for at least three studies (Crocetti 2016). Significance of moderators was tested with an omnibus test (i.e., the *Q*_m_ statistic) (Cheung 2014). Dummy variables were created for categorical moderators and follow up contrasts were examined to analyze differences in correlations between categories.

#### Publication bias

The presence of publication bias was examined by funnel plots, which show the relation between sample size and effect size. Funnel plots that imply no publication bias demonstrate symmetrical distributed ESs around the mean ES (Duval and Tweedie 2000). The symmetry of the funnel plot was examined by Eggers regression tests, in which sampling variance was added as a moderator to take into account the dependency between correlations from the same study (Egger et al. 1997). Because the correlation between empathy and the negative dimension of peer relationship quality was only examined in three studies, sampling variance could not be entered as a moderator, and therefore Egger’s regression test was performed without it. In case of funnel plot asymmetry, an adjusted correlation by using the “trim-and-fill” method was provided (Duval and Tweedie 2000). In addition to the funnel plots, the statistic Rosenthal’s *Fail-safe N* was calculated, which indicates how many missing studies would be required in order to find a non-significant (*p* > 0.05) correlation (Borenstein et al. 2011; Rosenthal 1979). Data of aggregated correlations at sample level (i.e., correlations averaged within samples) was used to examine the *Fail-safe N* with independent data.

## Results

### Study Sample

Because only two eligible studies were retrieved that examined sibling relationship quality and empathy, these two studies were excluded from the analyses (Lam et al. 2012; Harper et al. 2016)^3^.

In total, 390 effect sizes (ESs) of 70 studies (*n*) were retrieved, which included 75 independent samples (*n*_*sa*_). Specifically, 327 ESs from 57 independent samples (*N* = 28951^4^) were retrieved of the association between parent–child relationship quality and empathy, and 63 ESs from 31 independent samples (*N* = 25423^2^) of the association between peer relationship quality and empathy.

Concerning the 75 independent samples, sample sizes ranged from 39 to 7052 (*M* = 614, *SD* = 1024), mean ages from 10.5 to 20.0 years old (*M* = 15.0, *SD* = 2.3), and percentage of girls from 0% to 100% (*M* = 52.4%, *SD* = 27.8). Participants were from the continents America (*n*_*sa*_*=* 33), Europe (*n*_*sa*_ = 26), Asia (*n*_*sa*_*=* 9), Australia (*n*_*sa*_*=* 1), Oceania (*n*_*sa*_*=* 1), a mix of continents (*n*_*sa*_*=* 2), or unknown (*n=* 3). Of the 70 studies, 37 examined a sample in which the participants were from the ethnic majority. Only three examined samples were mainly composed of ethnic minorities, and 9 studies examined a mixed sample. Fourteen studies did not report on participant ethnicity. The countries of publication were mainly United States (*n* = 39), the Netherlands (*n* = 7), United Kingdom (*n* = 3), Italy (*n* = 3), and China (*n* = 3). Publication year of the studies ranged from 1982 to 2018, with a mean of 2010 and a median of 2012.

### Association between Relationship Quality and Adolescent Empathy

A small positive correlation of 0.18 (95% CI [0.15, 0.20], *p* < 0.001) was found between parent–child relationship quality and empathy, and a small-to-moderate correlation of 0.29 (95% CI [0.24, 0.33], *p* < 0.001) between peer relationship quality and empathy. With respect to the separate relationship quality dimensions, a small positive correlation with empathy was found for the positive dimension of parent–child relationship quality (*r* = 0.19, 95% CI [0.16, 0.22], *p* < 0.001) and a moderate positive correlation for the positive dimension of peer relationship quality (*r* = 0.31, 95% CI [0.26, 0.35], *p* < 0.001). Small negative correlations with empathy were found for the negative dimension of parent–child relationship quality (*r* = −0.13, 95% CI [−0.18, −0.07], *p* < 0.001) and peer relationship quality (*r* = −0.11, 95% CI [−0.17, −0.06], *p* < 0.001). Moreover, the overall correlation with peer relationship quality was significantly stronger than the correlation with parent–child relationship quality, *F*(1, 388) = 13.74, *p* < 0.001.

### Test of Heterogeneity

Concerning the correlation between parent–child relationship quality and empathy, the significant *Q*-statistic (*Q*(326) = 2197.46, *p* < 0.001) suggested variance between correlations. Constraining the within-study variance (*χ*^2^(1) = 512.17, *p* < 0.001) and the between-study variance to zero (*χ*^2^ (1) = 43.69, *p* < 0.001) resulted in a deteriorated model fit, suggesting that the variances deviated from zero. The distribution of variance across the three levels was: 14.6% (sampling variance), 53.6% (within-study variance), and 31.8% (between-study variance).

There was also significant variance between correlations for peer relationship quality and empathy (*Q*(62) = 1023.52, *p* < 0.001). Constraining the variances within studies (*χ*^2^(1) = 50.50, *p* < 0.001) and between studies to zero (*χ*^2^(1) = 7.06, *p* = 0.008) resulted in worse model fits, indicating that the variances differed significantly from zero. The distribution of variance across the three levels was: 2.0% (sampling variance), 44.1% (within-study variance), and 54.0% (between-study variance).

### Moderator Analyses

Results of the moderator analyses are reported in Table 2 (for parent–child relationship quality) and Table 3 (for peer relationship quality). In the tables, the number of effect sizes (*k*), number of samples (*n*_sa_), correlation coefficient (*r*) or slope, and the statistic of the moderator test (*Q*_m_) with its *p*-value are reported.

**Table 2:** Results of the Association between Parent–Child Relationship Quality and Empathy

Variable	*k*	*n*_sa_	*r/*slope	95% CI		*Q*_M_	*p*-value
				Lower	Upper		
Overall correlation	327	57	0.18***	0.15	0.20		
Moderator analyses							
Dimensions							
Dimension RQ	327	57				*F*(1, 325) = 120.13	< 0.001
Positive	186	53	0.20^a^***	0.18	0.23		
Negative (reverse coded)	141	10	0.05^b^**	0.01	0.08		
Positive indicator RQ	180	48				*F*(2, 177) = 0.21	0.812
Warmth	100	25	0.20***	0.16	0.24		
Attachment	35	19	0.20***	0.15	0.25		
Quality of communication	45	9	0.18***	0.12	0.24		
Dimension empathy	327	57				*F*(2, 324) = 3.71	0.026
Affective	181	37	0.16^a^***	0.13	0.18		
Cognitive	126	22	0.17^ab^***	0.14	0.21		
Composite	20	15	0.24^b^***	0.18	0.29		
Indicator empathy	282	47				*F*(4, 277) = 2.20	0.069
Empathic concern	132	21	0.17^a^***	0.13	0.21		
Composite affective	22	7	0.17^ab^***	0.10	0.24		
Mentalizing	8	3	0.11^ab^*	0.00	0.22		
Perspective taking	100	14	0.20^ab^***	0.16	0.23		
Composite EC/PT	20	16	0.24^b^***	0.18	0.29		
Type of relationship							
Type of parent–child relationship	327	57				*F*(2, 324) = 5.89	0.003
No distinction (parents)	69	29	0.21^a^***	0.17	0.25		
Mother	136	28	0.17^a^***	0.13	0.20		
Father	122	24	0.14^b^***	0.10	0.17		
Sample and study characteristics							
Age	302	54				*F*(1, 300) = 0.00	0.997
Intercept (centered)			0.18***	0.16	0.21		
Slope age			0.00	−0.01	0.01		
% Girls	314	51				*F*(1, 312) = 0.35	0.553
Intercept			0.19***	0.14	0.24		
Slope			0.00	−0.00	0.00		
Ethnic composition	277	34				*F*(1, 275) = 0.41	0.523
Majority	253	29	0.15***	0.12	0.19		
Mixed	24	6	0.18***	0.11	0.25		
Publication year (centered)	327	57				*F*(1, 325) = 2.81	0.095
Intercept			0.18***	0.15	0.21		
Slope			0.02	0.00	0.05		
Measurement characteristics							
Reliability RQ (centered)	219	47				*F*(1, 217) = 34.63	< 0.001
Intercept			0.18***	0.15	0.21		
Slope			0.05***	0.03	0.06		
Reliability empathy (centered)	290	52				*F*(1, 288) = 0.69	0.409
Intercept			0.18***	0.16	0.21		
Slope			−0.01	−0.03	0.01		
Informant RQ	322	55				*F*(1, 320) = 0.58	0.448
Self	286	54	0.18***	0.15	0.20		
Parent	36	5	0.19***	0.15	0.24		
Informant empathy	319	57				*F*(1, 317) = 9.39	0.002
Self	297	54	0.18^a^***	0.16	0.21		
Observant	22	6	0.06^b^	−.01	0.14		
Assessment empathy	327	57				*F*(2, 324) = 4.07	0.018
Questionnaire	298	51	0.19^a^***	0.17	0.22		
Observation	14	4	0.07^b^*	−0.03	0.16		
Task	15	4	0.11^ab^*	0.02	0.20		
Questionnaire empathy	292	48				*F*(3, 288) = 1.45	0.229
IRI	244	31	0.20^a^***	0.17	0.23		
IECA	23	7	0.18^ab^***	0.12	0.24		
BES	5	4	0.20^ab^***	0.09	0.30		
Emotional empathy scale	20	6	0.11^b^*	0.02	0.18		

Estimates with different subscripts differed significantly in strength

*k* number of correlations, *n*_*sa*_ number of samples, *RQ* relationship quality, *Composite EC/PT* combination empathic concern and perspective taking, *IRI* Interpersonal Reactivity Index, *IECA* Index of Empathy for Children and Adolescents, *BES* Basic Empathy Scale

*p < 0.05, **p < 0.01, ***p < 0.001

**Table 3:** Results of the Association between Peer Relationship Quality and Empathy

Variable	*k*	*n*_sa_	*r/*slope	95% CI		*Q*_M_	*p*-value
				Lower	Upper		
Overall correlation	63	31	0.29***	0.24	0.33		
Moderator analyses							
Dimensions							
Dimension RQ	63	31				*F*(1, 61) = 13.90	< 0.001
Positive	49	30	0.30^a^***	0.26	0.35		
Negative (reverse coded)	14	3	0.15^b^**	0.05	0.24		
Positive indicator RQ	21	18				*F*(2, 18) = 0.31	0.738
Warmth	6	5	0.39***	0.28	0.49		
Attachment	9	9	0.35***	0.26	0.43		
Friendship quality	6	4	0.40***	0.26	0.52		
Dimension empathy	63	31				*F*(2, 60) = 6.32	0.003
Affective	23	12	0.23^a^***	0.16	0.28		
Cognitive	27	14	0.24^a^***	0.18	0.30		
Composite	13	13	0.38^b^***	0.31	0.44		
Indicator empathy	45	22				*F*(2, 42) = 5.88	0.006
Empathic concern	19	9	0.22^a^***	0.15	0.28		
Perspective taking	14	7	0.26^a^***	0.18	0.34		
Composite EC/PT	12	12	0.38^b^***	0.31	0.44		
Type of relationship							
Type peer relationship	63	31				*F*(2, 60) = 2.38	0.101
Peer	3	3	0.36***	0.21	0.49		
Friend	49	23	0.30***	0.25	0.35		
Partner	11	6	0.20***	0.10	0.30		
Sample characteristics							
Age	61	30				*F*(1, 59) = 3.91	0.053
Intercept (centered)			0.31***	0.26	0.36		
Slope age			−0.02	−0.05	0.00		
% girls	63	31				*F*(1, 61) = 0.01	0.907
Intercept			0.28***	0.19	0.37		
Slope			0.00	−0.00	0.00		
Ethnicity	44	19				*F*(2, 41) = 5.13	0.010
Majority	33	10	0.40^a^***	0.31	0.49		
Minority	4	3	0.30^ab^***	0.15	0.43		
Mixed	7	6	0.22^b^***	0.15	0.28		
Publication year	51	20				*F*(1, 61) = 0.00	0.995
Intercept (centered)			0.29***	0.24	0.34		
Slope			0.00	−0.05	0.05		
Measurement characteristics							
Reliability RQ	46	26				*F*(1, 44) = 20.39	< 0.001
Intercept (centered)			0.30***	0.25	0.35		
Slope			0.09***	0.05	0.14		
Reliability empathy	40	25				*F*(1, 38) = 7.68	0.009
Intercept (centered)			0.28***	0.23	0.33		
Slope			0.07**	0.02	0.12		

Estimates with different subscripts differed significantly in strength

*k* number of correlations, *n*_*sa*_ number of samples, *RQ* relationship quality, *Composite EC/PT* combination empathic concern and perspective taking

*p < 0.05, **p < 0.01, ***p < 0.001

#### Relationship dimension

For both parent–child (*F*(1, 325) = 120.13, *p* < 0.001) and peer relationship quality (*F*(1, 61) = 13.90, *p* < 0.001), relationship quality dimension was a significant moderator, such that correlations with empathy were stronger for the positive dimensions of relationship quality compared to the negative dimension.

Correlations for separate indicators of the positive dimension (warmth vs. attachment vs. quality of communication/friendship quality) were not significantly different from each other for both parent–child (*F*(2, 177) = 0.21, *p* = 0.812) and peer relationship quality (*F*(2, 18) = 0.31, *p* = 0.738).

#### Empathy dimension

For both parent–child (*F*(2, 324) = 3.71, *p* = 0.026) and peer relationship quality (*F*(2, 60) = 6.32, *p* = 0.003), empathy dimension was a significant moderator. Follow up contrasts suggested that a composite score of affective and cognitive empathy was more strongly correlated to parent–child relationship quality than a separate score affective empathy (not cognitive empathy). Peer relationship quality was most strongly correlated to a composite score of affective and cognitive empathy then separate scores of both affective and cognitive empathy.

Specific indicator of empathy (e.g., empathic concern, perspective taking, mentalizing) did not moderate the correlation with parent–child relationship quality (*F*(4, 277) = 2.20, *p* = 0.069) but did moderate the correlation with peer relationship quality (*F*(2, 42) = 5.88, *p* = 0.006). However, follow up contrasts indicated a stronger correlation between parent–child relationship quality and a composite score of empathic concern and perspective taking compared to a separate score of empathic concern. For peer relationship quality, a stronger correlation was found with a composite score of empathic concern and perspective taking compared to separate scores of empathic concern or perspective taking.

#### Type of relationship

Type of relationship (parents vs. mother vs. father, or peer vs. friend vs. romantic partner) moderated the correlation with parent–child relationship quality (*F*(2, 324) = 5.89, *p* = 0.003) but not the correlation with peer relationship quality (*F*(2, 60) = 2.38, *p* = 0.101). Follow up contrasts indicated that the correlation of empathy was stronger with parent–child or mother–child relationship quality compared to father–child relationship quality.

#### Age and gender

Age and gender did not significantly moderate the correlations of empathy with parent–child (age: *F*(1, 300) = 0.00, *p* = 0.997; gender: *F*(1, 312) = 0.35, *p* = 0.553) and peer relationship quality (age: *F*(1, 59) = 3.91, *p* = 0.053; gender: *F*(1, 61) = 0.01, *p* = 0.907).

#### Additional sample, study, and measurement characteristics

With respect to ethnic composition (majority vs. minority vs. mixed), a significant moderation effect was found for the correlation between peer relationship quality and empathy (*F*(2, 41) = 5.13, *p* = 0.010), but not for the correlation between parent–child relationship quality and empathy (*F*(1, 275) = 0.41, *p* = 0.523). The results of the contrast analysis showed that a stronger correlation between peer relationship quality and empathy was found in majority samples compared to mixed samples^5^. However, correlations of minority samples did not differ from correlations of majority or mixed samples. Furthermore, publication year did not significantly moderate the correlations (parent–child: *F*(1, 325) = 2.81, *p* = 0.095; peer: *F*(1, 61) = 0.00, *p* = 0.995). Reliability of the relationship quality measurement moderated both correlations with parent–child (*F*(1, 217) = 34.63, *p<* 0.001) and peer relationship quality (*F*(1, 44) = 20.39, *p* < 0.001), such that stronger correlations were found with empathy when reliability of relationship quality was higher. Reliability of empathy measurement only moderated the correlation with peer relationship (*F*(1, 38) = 7.68, *p* = 0.009) and not with parent–child relationship quality (*F*(1, 288) = 0.69, *p* = 0.409). A stronger correlation between peer relationship quality and empathy was found when reliability of the empathy measurement was higher. The moderating roles of informant of relationship quality and empathy and assessment method of empathy could only be tested for the correlation with parent–child relationship quality, because of a lack of variety in the peer relationship studies (mainly based on self-reported questionnaires). The results demonstrated that informant of parent–child relationship quality (self- vs. parent-reported) was not a significant moderator (*F*(1, 320) = 0.58, *p* = 0.448), but informant (self- vs. observant-reported) (*F*(1, 317) = 9.39, *p* = 0.002) and assessment method of empathy (questionnaire vs. observation vs. task) (*F*(2, 324) = 4.07, *p* = 0.018) were significant moderators. Correlations were stronger when empathy was self-reported instead of observer-reported. Similarly, a stronger correlation was found when empathy was measured with a questionnaire compared to an observation. The correlation based on an empathy task (e.g., Reading the Mind in the Eyes Task) did not differ from the correlation based on a questionnaire measurement of empathy. Additionally, empathy questionnaire was not a significant moderator in the correlation between parent–child relationship quality and empathy (*F*(3, 288) = 1.45, *p* = 0.229). Nonetheless, contrast analysis showed a stronger correlation when the Interpersonal Reactivity Index was used compared to the Mehrabian and Epstein’s Emotional Empathy Scale. The correlation based on the Emotional Empathy Scale did not differ from the correlations based on the Empathy Index for Children and Adolescents or the Basic Empathy Scale.

### Sensitivity Analysis

The positive and negative dimensions of relationship quality can be considered as distinct constructs, such that lower levels of negativity might not by definition represent higher relationship quality. Therefore, sensitivity analyses were conducted to check whether results of the previous described moderator analyses were different when data of the positive and negative relationship quality dimensions were separately analyzed and the effect sizes of the negative dimension were not reverse coded (thus original effect size, with negative correlations representing higher levels of negativity and lower levels of empathy). For peer relationship quality, moderator analysis on the separate dimensions could only be performed with the *positive dimension*, as not enough studies were present for the *negative dimension* (three studies) to run moderator analyses. Results of the sensitivity analyses are displayed in Online Resources 1, 2, and 3.

With respect to hypothesized moderators, two differences were found when comparing results of the moderator analyses performed on the complete correlational data or on the data of the separate relationship quality dimensions. First, when examining empathy indicator as a moderator in the association between the *positive dimension* of parent–child relationship quality and empathy, the correlations with empathic concern and perspective taking became different in strength. Such that the correlation with perspective taking was stronger than the correlation with empathic concern. Second, when examining type of parent–child relationship as a moderator in the association between the *negative dimension* of parent–child relationship quality and empathy, the correlations for mothers and father were not different in strength anymore.

### Testing Publication Bias

The results of the Egger’s regression test demonstrated funnel plot asymmetry for (1) the correlation between parent–child relationship quality and affective empathy, (2) the overall correlation between peer relationship quality and empathy, and (3) the correlation between the positive dimension of peer relationship quality and empathy (see Table 4). After using the trim and fill method, a slightly weaker correlation was found between parent–child relationship quality and affective empathy (*r=* 0.17, *p* < 0.001, 95% CI [0.14, 0.20], and stronger correlations were found for the overall correlation peer relationship quality and empathy (*r* = 0.35, *p* < 0.001, 95% CI [0.30, 0.39]) and between the positive dimension of peer relationship quality and empathy (*r* = 0.36, *p* < 0.001, 95% CI [0.31, 0.40]). Furthermore, according to Rosenthal’s criterion (*n* × 5 + 10) (Rosenthal 1995), results of the Fail-safe method indicated that the correlation between the negative dimension of peer relationship quality and empathy is likely an artifact of publication bias (see Table 4).

**Table 4:** Tests for Publication Bias

	Parent–child relationship quality and empathy			
	Funnel plot symmetry			Fail-safe *N*
	*z*-value	*p*-value		
Relationship quality	0.92	0.356		16729
Positive	0.93	0.354		15986
Negative	1.91	0.055		247
Empathy	0.92	0.356		16729
Affective	2.44	0.015		4365
Cognitive	−0.52	0.603		2167
Composite	0.19	0.851		3398

	Peer relationship quality and empathy			
	Funnel plot symmetry			Fail-safe *N*
	*z*-value	*p*-value		
Relationship quality	−2.62	0.009	23453	
Positive	−1.86	0.062	23685	
Negative	0.54	0.586	11	
Empathy	−2.62	0.009	23453	
Affective	0.12	0.904	1026	
Cognitive	−1.47	0.141	2265	
Composite	−0.62	0.536	9750	

## Discussion

Empathy, or the ability to feel concern for and to understand others’ feelings, is thought to develop in high quality relationships with parent and peers, but also to facilitate the quality of these relationships. Studies on adolescent relationship quality and empathy have assessed a variety of relationship quality indicators (e.g., support, conflict, satisfaction) and have made a distinction between affective and cognitive empathy. Therefore, uncertainty remained about how parent–child and peer relationship quality were associated with empathy in adolescence. In order to enhance the understanding of how these constructs are associated, the present meta-analysis was conducted. The meta-analysis applied a multilevel approach in order to include multiple effect sizes from one study or sample, thus allowing to extract all available empirical data. In total, 390 effect sizes from 70 studies, assessing 75 independent samples, were retrieved. In line with hypotheses, the results indicated that both parent–child and peer relationship quality were positively associated with adolescent empathy. However, as expected, empathy was more strongly associated with peer relationship quality than with parent–child relationship quality. Moreover, although many moderators did not appear to affect the strength of the associations (e.g., age and gender), several significant moderators were identified, such as relationship quality dimension (positive vs. negative dimension) and gender of the parent (mother vs. father). Hence, the present meta-analysis demonstrates that adolescents with higher quality relationships, particularly with peers, have indeed more concern for and a better understand of others’ emotions.

### Parent–Child versus Peer Relationship Quality

The results of the meta-analysis confirmed that adolescents with higher quality parent–child and peer relationships have higher levels of empathy compared to adolescents with lower quality relationships. This is in line with socialization theories describing the facilitating role of supportive relationships in the development of empathy, for example by modelling (Barnett 1987), and the expectation that empathy promotes relationship quality through more constructive conflict behavior (e.g. Van Lissa et al. 2016). Furthermore, a stronger correlation of empathy with peer relationship quality than with parent–child relationship quality was found. This is in accordance with socialization theories positing a more influential role of peers compared to parents in adolescence (Youniss and Smoller 1985). Additionally, as adolescence is a period in which time spent with peers increases and peer relationships become more intimate (Furman and Buhrmester 1992; Larson and Richards 1991), empathy may be more important to gain and maintain high quality peer relationships than to maintain high quality parent–child relationships. However, since the meta-analytic results are based on correlational studies investigating relative inter-individual differences in adolescents’ empathy and relationship quality, it cannot be certain that these positive correlations also reflect over-time processes of facilitative effects of relationship quality on empathy or vice versa (Hamaker 2012). Nonetheless, this meta-analysis including 70 studies powerfully indicates that relative inter-individual differences in empathy and relationship quality, particularly regarding relationships with peers, are related in adolescence, and encourages future studies to investigate the underlying intra-individual processes (e.g., Lam et al. 2012) that may form these inter-individual differences.

### Explaining Heterogeneity with Moderators

#### Dimensions of relationship quality and empathy

As expected, the results showed that the positive dimensions of parent–child and peer relationship quality were positively related to empathy and the negative dimensions were negatively related to empathy in adolescence. Additionally, the correlations with the positive dimensions were stronger than with the negative dimensions. The finding that the positive dimension of relationship quality is stronger related to empathy than the negative dimension, can be explained by the conceptual connection between empathy and the positive dimension of relationship quality. That is, warmth and support in relationships with parents or peers imply concern and understanding for adolescents’ emotions and this provides the adolescent with a model of empathy. Nonetheless, it should be noted that only fourteen effect sizes from three studies could be included of the negative dimension of peer relationship quality, and hence future analyses involving more correlations can provide a more reliable conclusion regarding the association between negative peer relationship quality and empathy in adolescence.

Moreover, the dimensions affective and cognitive empathy were not differently related to parent–child and peer relationship quality in adolescence. However, when examining the positive and negative dimension of parent–child relationship quality separately, the positive dimension of parent–child relationship quality was more strongly related to perspective taking than to empathic concern. Nonetheless, the positive dimension of parent–child relationship quality was not differently related to a combination score of perspective taking and empathic concern compared to the separate scores of perspective taking or empathic concern. Hence, the meta-analytic findings were contrary to the hypotheses that affective empathy would most strongly be associated with parent–child relationship quality and cognitive empathy with peer relationship quality, which were based on socialization theories emphasizing different aspects of empathy. The findings suggest that adolescents who both show more concern for and a better understanding of others’ emotions experience higher quality relationships with parents and peers.

Additionally, a composite score of affective and cognitive responses was most strongly associated with parent–child and peer relationship quality compared to separate scores of affective and/or cognitive responses. One possible explanation is that empathy is more reliably measured when taking into account both affective and cognitive responses, for example, simply because the measure consists of more items. Another explanation is that being high on either cognitive empathy or affective empathy is not enough, but that it takes both to maintain higher quality relationships. Although affective and cognitive empathy were similarly related to relationship quality, they tap into different aspects of empathy that are both beneficial for relational functioning. This is in line with previous studies showing that perspective taking alone did not predict adolescents’ prosocial behavior, but that it predicted prosocial behavior either indirectly through its association with affective empathy (Van der Graaff et al. 2018) or in interaction with affective empathy (e.g., Eisenberg et al. 2001). Nonetheless, assessing the dimensions separately, but preferably simultaneously, is still of interest as this gives insight in how affective and cognitive empathy, and their combination, facilitate high quality relationships.

#### Type of parent–child and peer relationship

In line with the hypothesis, the moderation analyses showed that mother–child relationship quality was more strongly associated with empathy in adolescence than father–child relationship quality. As adolescent generally spend more time with their mothers than with their fathers (Yeung et al. 2001) and fathers are less aware of their children’s prosocial behavior (Hastings et al. 2007), the positive loop between constructive interpersonal behaviors within the relationship and adolescent empathy might be stronger in mother-adolescent dyads. This is also supported by the results of the sensitivity analyses suggesting that this is particularly true for the positive dimension of parent–child relationship quality and not for the negative dimension. Regarding relationship quality with friends versus romantic partners, correlations with empathy did not differ. However, it should be noted that only six samples from three studies assessing romantic relationship quality were included, whereas there were twenty-three samples from twenty studies assessing friendship quality. Furthermore, a comparison with sibling relationship quality could not be made as only two studies were available. Therefore, more studies on the associations of romantic relationship quality and sibling relationship quality with adolescent empathy are needed in order to draw a more robust conclusion about the (dis)similarity of the correlations of empathy with different types of peer relationships.

#### Age, gender, and additional sample, study, and measurement characteristics

It was expected that the associations of parent–child and peer relationship quality with empathy would differ in strength across adolescence and between boys and girls because of differences in socialization processes (e.g., Bem 1981) and changes within relationships across adolescence (Smetana et al. 2006). However, associations varied neither with age nor gender, and the strength of both associations of parent–child and peer relationship quality with empathy appeared rather robust in adolescence. Furthermore, some additional moderators were tested. Results indicated that the strength of the correlation between parent–child relationship quality and empathy was higher (I) when reliability of the parent–child relationship quality measurement was higher and (II) when empathy was measured with self-reported questionnaires compared to observational data. Concerning the association between peer relationship quality and empathy, analyses showed that the strength of this correlation was higher when (I) measured in mixed ethnic samples compared to majority samples and (II) reliability of peer relationship quality and empathy was higher. However, the correlations of the mixed samples were based on more reliable empathy data (average reliability of 0.81) compared to the correlations of the majority samples (average reliability of 0.72). After controlling for the reliability of the empathy measurements, correlations between peer relationship quality and empathy did not differ any longer for mixed and majority samples. Furthermore, the moderating effect of informant of the reports of relationship quality and empathy, and assessment method of relationship quality could not be tested in this association due to a lack of data. Thus, less reliable measures of both relationship quality and empathy appeared to be a factor that explained findings of less strong associations, but not enough variation in assessment method was present to assess this as a moderator for explaining heterogeneity in the association between peer relationship quality and empathy in adolescence.

### Strengths, Limitations, and Directions for Future Research

The present meta-analysis is the first to examine the associations between parent–child and peer relationship quality and empathy in adolescence. A multilevel meta-analysis approach (Van den Noortgate et al. 2015) enables to include multiple effect sizes within studies, allowing to incorporate all relevant data that was available. Moreover, a search in several relevant electronic databases was conducted by using a broad range of related key words. The comprehensive nature of the database search was confirmed by the fact that only found two additional studies were found through backwards citation search. Furthermore, a considerable number of moderators enabled to answer longstanding questions, such as differences between affective and cognitive empathy, and genders. However, the associations of empathy with parent–child and peer relationship quality appeared robust and significant across different types of relationships, dimensions of empathy, age and gender, suggesting that empathy and relationship are consistently related in adolescence.

Notwithstanding these strengths, some limitations should be considered. First, only 26 of the 70 included studies examined the association between peer relationship quality and empathy. Of these studies, only 14 effect sizes were retrieved that examined the association between the negative dimension of peer relationship quality and adolescent empathy. As a consequence, the estimation of the true effect size linking peer relationship quality and empathy is less precise than the estimation for the association between the negative dimension of parent–child relationship quality and empathy. Moreover, it was impossible to include sibling relationship quality due to a lack of eligible studies. Hence, it is recommended that future studies focus on the role of peer relationship quality, and in particular relationships with siblings, in adolescent empathy. Second, the investigated associations were concurrent, and therefore, findings of the present study cannot be translated to longitudinal effects. To the best of our knowledge, the literature predominantly consists of cross-sectional studies, and hence, it is recommended that future studies consider longitudinal associations between empathy and relationship quality. Third, no conclusions can be drawn with respect to intra-individual processes that occur over time, as correlations were examined that demonstrate cross-sectional relative differences between adolescents in relationship quality and empathy. Conducting longitudinal within-person studies is an important next step to assess the reciprocal intra-individual processes between relationship quality and empathy in adolescence.

## Conclusion

The present meta-analysis investigated the associations between parent–child and peer relationship quality and empathy in adolescents. Because empirical studies on this topic varied greatly in design, ambiguity remained about how relationship quality with parents and peers is associated with adolescent empathy. In order to provide a better comprehension of these associations, the present meta-analysis applied a multilevel approach to synthesize all available data. As expected, the results demonstrated that adolescents with higher quality parent–child and peer relationships show more concern for and a better understanding of others’ emotions than adolescents with poorer parent–child and peer relationships. These associations appeared rather robust. Additionally, the moderator analyses indicated some significant moderators that explained differences in strength, such as relationship quality dimension and type of parent–child relationship. To conclude, the findings imply that good empathic abilities may be protective for experiencing poor relationships and that higher quality relationships might facilitate the development of empathic skills. As poor interpersonal functioning is related to maladaptation, such as heightened levels of depressive feelings (e.g., Zhang et al. 2018), the present study contributes to an improved comprehension of protective factors of poor interpersonal functioning during adolescence.

## Supplementary information

Online Resources

## Footnotes

1 Socioeconomic status (SES) was not included as a moderator, because information about SES was too diverse across articles or was lacking, and therefore difficult to compare between studies. Initially, the first and second author coded SES in a subset of articles, but inter-rater reliability was low, and therefore SES was dropped as a moderator.

2 When authors only reported the school grades of the participants, a mean age that was likely the mean age of the sample (10 years for fifth grade and 17 years for twelfth grade) was calculated. For example, when participants were in the fifth grade (10 years) and sixth grade (11 years), a mean age of 10.5 was attributed.

3 Harper et al. (2016) reported significant correlations between sibling affection and affective empathy of 0.22 for boys and 0.29 for girls, and nonsignificant correlations between sibling conflict and affective empathy of −0.01 for both boys and girls. The data of Lam et al. (2012) showed significant correlations between sibling warmth and affective empathy of 0.24 for the youngest siblings and 0.42 for the oldest sibling, and a nonsignificant correlation between sibling conflict and affective empathy of −0.08 for the youngest sibling and a significant correlation of −0.18 for the oldest sibling.

4 When multiple studies examined the same sample, the size of the largest sample was counted.

5 After controlling for reliability of empathy measures, correlations did not differ anymore for majority, minority, and mixed samples.
