# Supplementary material for: Linking Parent–Child and Peer Relationship Quality to Empathy in Adolescence: A Multilevel Meta-Analysis
Source: J Youth Adolesc. 2019 Feb 27;48(6):1033–55. doi: 10.1007/s10964-019-00993-5 (PMC6525137; doi:10.1007/s10964-019-00993-5)
Supplement: Supplementary file 1 — Online Resources [file 10964_2019_993_MOESM1_ESM.docx]

Online Resource 1

*Moderator Analyses on the Association between the Positive Dimension of Parent-Child Relationship Quality and Empathy*

| Variable | *k* | *n*_sa_ | *r /*slope | 95% CI | | *Q*_M_ | *p*-value |
| --- | --- | --- | --- | --- | --- | --- | --- |
|  |  |  |  | Lower | Upper |  |  |
| *Dimensions* |  |  |  |  |  |  |  |
| Dimension empathy | 186 | 53 |  |  |  | *F*(2, 183) = 3.90 | .022 |
| Affective | 110 | 35 | .17^a***^ | .13 | .20 |  |  |
| Cognitive | 58 | 20 | .20^ab***^ | .16 | .23 |  |  |
| Composite | 18 | 15 | .24^b***^ | .19 | .30 |  |  |
| Indicator empathy | 144 | 41 |  |  |  | *F*(3, 140) = 3.05 | .031 |
| Empathic concern | 66 | 20 | .18^a***^ | .13 | .22 |  |  |
| Composite affective | 22 | 7 | .19^ab***^ | .11 | .26 |  |  |
| Perspective taking | 38 | 14 | .22^b***^ | .18 | .27 |  |  |
| Composite EC/PT | 18 | 15 | .25^ab***^ | .19 | .30 |  |  |
| *Type of relationship* |  |  |  |  |  |  |  |
| Type of parent-child relationship | 186 | 53 |  |  |  | *F*(2, 183) = 10.50 | < .001 |
| No distinction (parents) | 54 | 28 | .21^a^*** | .17 | .25 |  |  |
| Mother | 73 | 25 | .20^a^*** | .16 | .24 |  |  |
| Father | 59 | 21 | .14^b^*** | .10 | .18 |  |  |
| *Sample and study characteristics* |  |  |  |  |  |  |  |
| Age | 161 | 50 |  |  |  | *F*(1, 159) = 0.18 | .671 |
| Intercept (centered) |  |  | .20*** | .17 | .23 |  |  |
| Slope age |  |  | .00 | -.01 | .01 |  |  |
| % Girls | 173 | 47 |  |  |  | *F*(1, 171) = 0.17 | .682 |
| Intercept |  |  | .20*** | .15 | .26 |  |  |
| Slope |  |  | .00 | .00 | .00 |  |  |
| Ethnic composition | 145 | 31 |  |  |  | *F*(1, 143) = 0.19 | .661 |
| Majority | 123 | 27 | .17*** | .13 | .21 |  |  |
| Mixed | 22 | 5 | .19*** | .11 | .26 |  |  |
| Publication year (centered) | 186 | 53 |  |  |  | *F*(1, 184) = 5.88 | .016 |
| Intercept |  |  | .18*** | .16 | .21 |  |  |
| Slope |  |  | .04* | .01 | .08 |  |  |
| *Measurement characteristics* |  |  |  |  |  |  |  |
| Reliability RQ (centered) | 130 | 44 |  |  |  | *F*(1, 128) = 0.20 | .654 |
| Intercept |  |  | .19*** | .17 | .22 |  |  |
| Slope |  |  | .01 | -.02 | .03 |  |  |
| Reliability empathy (centered) | 156 | 50 |  |  |  | *F*(1, 154) = 1.78 | .184 |
| Intercept |  |  | .20*** | .17 | .23 |  |  |
| Slope |  |  | -.02 | -.04 | .01 |  |  |
| Informant RQ | 182 | 52 |  |  |  | *F*(1, 180) = 5.37 | .022 |
| Self | 170 | 51 | .20^a^*** | .17 | .23 |  |  |
| Parent | 12 | 4 | .12^b^*** | .05 | .19 |  |  |
| Informant empathy | 178 | 53 |  |  |  | *F*(1, 176) = 5.37 | .003 |
| Self | 157 | 50 | .20^b^*** | .18 | .23 |  |  |
| Observant | 21 | 6 | .08^b^* | .01 | .16 |  |  |
| Assessment empathy | 176 | 51 |  |  |  | *F*(1, 174) = 6.76 | .010 |
| Questionnaire | 165 | 49 | .20^a^*** | .18 | .23 |  |  |
| Observation | 13 | 4 | .08^b^ | -.02 | .17 |  |  |
| Questionnaire empathy | 157 | 46 |  |  |  | *F*(3, 153) = 2.19 | .092 |
| IRI | 114 | 29 | .22^a^*** | .19 | .25 |  |  |
| IECA | 18 | 7 | .20^ab^*** | .13 | .27 |  |  |
| BES | 5 | 4 | .20^ab^*** | .10 | .29 |  |  |
| Emotional empathy scale | 20 | 6 | .11^b^* | .02 | .19 |  |  |

*Note*. Different subscripts indicate that the correlations significantly differed in strength. *k* = number of correlations. *n*_sa_ = number of samples. RQ = relationship quality. Composite EC/PT = combination empathic concern and perspective taking. IRI = Interpersonal Reactivity Index. IECA = Index of Empathy for Children and Adolescents. BES = Basic Empathy Scale.
**p* < .05, ***p* < .01, ****p* < .001.

Online Resource 2

*Moderator Analyses on the Association between the Negative Dimension of Parent-Child Relationship Quality and Empathy*

| Variable | *k* | *n*_sa_ | *r /*slope | 95% CI | | *Q*_M_ | *p*-value |
| --- | --- | --- | --- | --- | --- | --- | --- |
|  |  |  |  | Lower | Upper |  |  |
| *Dimensions* |  |  |  |  |  |  |  |
| Dimension empathy | 139 | 10 |  |  |  | *F*(1, 37) = 2.90 | .090 |
| Affective | 71 | 7 | -.11*** | -.17 | -.05 |  |  |
| Cognitive | 68 | 7 | -.13 | -.19 | -.07 |  |  |
| Indicator empathy | 134 | 8 |  |  |  | *F*(2, 131) = 1.44 | .241 |
| Empathic concern | 66 | 5 | -.11** | -.18 | -.04 |  |  |
| Perspective taking | 62 | 4 | -.13*** | -.20 | -.06 |  |  |
| Mentalizing | 6 | 3 | -.17** | -.29 | -.04 |  |  |
| *Type of relationship* |  |  |  |  |  |  |  |
| Type of parent-child relationship | 141 | 11 |  |  |  | *F*(2, 138) = 0.50 | .610 |
| No distinction (parents) | 15 | 5 | -.16*** | -.24 | -.07 |  |  |
| Mother | 65 | 6 | -.11*** | -.18 | -.05 |  |  |
| Father | 63 | 6 | -.11** | -.18 | -.05 |  |  |
| *Sample and study characteristics* |  |  |  |  |  |  |  |
| Age | 141 | 11 |  |  |  | *F*(1, 139) = 0.18 | .786 |
| Intercept (centered) |  |  | -.13*** | -.18 | -.07 |  |  |
| Slope age |  |  | .00 | -.01 | .01 |  |  |
| % Girls | 141 | 11 |  |  |  | *F*(1, 139) = 0.72 | .397 |
| Intercept |  |  | -.17** | -.27 | -.06 |  |  |
| Slope |  |  | .00 | .00 | .00 |  |  |
| Publication year (centered) | 141 | 11 |  |  |  | *F*(1, 139) = 29.28 | < .001 |
| Intercept |  |  | .06 | -.10 | .21 |  |  |
| Slope |  |  | .13*** | .08 | .17 |  |  |
| *Measurement characteristics* |  |  |  |  |  |  |  |
| Reliability RQ (centered) | 89 | 10 |  |  |  | *F*(1, 87) = 91.49 | < .001 |
| Intercept |  |  | -.25*** | -.33 | -.16 |  |  |
| Slope |  |  | -.10*** | -.12 | -.08 |  |  |
| Reliability empathy (centered) | 134 | 9 |  |  |  | *F*(1, 132) = 0.74 | .393 |
| Intercept |  |  | -.12*** | -.18 | -.06 |  |  |
| Slope |  |  | -.01 | -.02 | .01 |  |  |

*Note*. Different subscripts indicate that the correlations significantly differed in strength. *k* = number of correlations. *n*_sa_ = number of samples. RQ = relationship quality.
**p* < .05, ***p* < .01, ****p* < .001.

Online Resource 3

*Moderator Analyses on the Association between the Positive Dimension of Peer Relationship Quality and Empathy*

| Variable | *k* | *n*_sa_ | *r /*slope | 95% CI | | *Q*_M_ | *p*-value |
| --- | --- | --- | --- | --- | --- | --- | --- |
| *Dimensions* |  |  |  |  |  |  |  |
| Dimension empathy | 49 | 30 |  |  |  | *F*(2, 46) = 6.08 | .005 |
| Affective | 18 | 12 | .24^a^*** | .18 | .29 |  |  |
| Cognitive | 18 | 13 | .28^a^*** | .22 | .33 |  |  |
| Composite | 13 | 13 | .38^b^*** | .31 | .43 |  |  |
| Indicator empathy | 37 | 22 |  |  |  | *F*(2, 34) = 5.76 | .007 |
| Empathic concern | 15 | 9 | .22^a^*** | .15 | .29 |  |  |
| Perspective taking | 10 | 7 | .29^ab^*** | .21 | .37 |  |  |
| Composite EC/PT | 12 | 12 | .38^b^*** | .31 | .44 |  |  |
| *Type of relationship* |  |  |  |  |  |  |  |
| Type peer relationship | 49 | 30 |  |  |  | *F*(2, 46) = 1.46 | .244 |
| Peer | 3 | 3 | .36*** | .22 | .48 |  |  |
| Friend | 37 | 22 | .32*** | .26 | .37 |  |  |
| Partner | 9 | 5 | .23*** | .12 | .33 |  |  |
| *Sample characteristics* |  |  |  |  |  |  |  |
| Age | 47 | 29 |  |  |  | *F*(1, 45) = 4.05 | .050 |
| Intercept (centered) |  |  | .32*** | .27 | .37 |  |  |
| Slope age |  |  | -.02 | -.04 | .00 |  |  |
| % girls | 49 | 30 |  |  |  | *F*(1, 47) = 0.00 | .988 |
| Intercept |  |  | .31*** | .22 | .39 |  |  |
| Slope |  |  | .00 | .00 | .00 |  |  |
| Ethnicity | 34 | 19 |  |  |  | *F*(2, 31) = 3.80 | .033 |
| Majority | 23 | 10 | .41^a^*** | .31 | .50 |  |  |
| Minority | 4 | 3 | .30^ab^*** | .14 | .44 |  |  |
| Mixed | 7 | 6 | .24^b^*** | .17 | .31 |  |  |
| Publication year | 49 | 30 |  |  |  | *F*(1, 47) = 0.01 | .911 |
| Intercept (centered) |  |  | .31*** | .26 | .35 |  |  |
| Slope |  |  | .00 | -.05 | .05 |  |  |
| *Measurement characteristics* |  |  |  |  |  |  |  |
| Reliability RQ | 41 | 26 |  |  |  | *F*(1, 39) = 8.68 | .005 |
| Intercept (centered) |  |  | .31*** | .26 | .35 |  |  |
| Slope |  |  | .07** | .02 | .12 |  |  |
| Reliability empathy |  |  |  |  |  | *F*(1, 32) = 5.76 | .022 |
| Intercept (centered) |  |  | .31*** | .26 | .36 |  |  |
| Slope |  |  | .06* | .01 | .11 |  |  |

*Note* Different subscripts indicate that the correlations significantly differed in strength. *k* = number of correlations. *n*_sa_ = number of samples. RQ = relationship quality. Composite EC/PT = combination empathic concern and perspective taking.  
**p* < .05, ***p* < .01, ****p* < .001.
